# The Implications of Ungulate Population Dynamics for Conservation and Recovery Efforts in the Middle East

**DOI:** 10.1002/ece3.74428

**Published:** 2026-09-28

**Authors:** Ahmed S. Aljohani, Michael B. Bonsall, Robert A. Montgomery

**Affiliations:** ^1^ Department of Biology University of Oxford Oxford UK; ^2^ Institute of Science and Environment University of Cumbria Ambleside UK

**Keywords:** Allee effect, Arabian oryx ( *Oryx leucoryx* ), density dependence, reintroduction, sand gazelle (*Gazella marica*), ungulate conservation

## Abstract

Ungulate reintroduction programmes are increasingly implemented in the Middle East to counter substantial population declines attributed to overhunting and habitat degradation. Many of these populations, however, are highly conservation‐dependent, and the mechanisms regulating these populations remain largely unexamined. We compiled and analysed 84 oryx and 43 gazelle annual population records across the Middle East for the Arabian oryx ( *Oryx leucoryx* ) and sand gazelle (*Gazella marica*), both classified as Vulnerable on the IUCN Red List, using linear mixed‐effects models to test for patterns in population regulation focusing on density dependence, Allee effects, and temporal autocorrelation in growth rates. Both species showed a negative density dependence pattern, with growth rates declining consistently as population size increased, indicating that density dependence is an important driver of population persistence and dynamics across sites. We detected no significant Allee effects, suggesting that small populations can recover when conditions are suitable. The models of Arabian oryx exhibited temporal autocorrelation, with prior‐year conditions significantly influencing present growth trends, indicating that population dynamics are shaped by lagged environmental or demographic processes. Inconsistent monitoring limited our ability to assess other ungulate species in the region, highlighting the need for more standardised long‐term data collection across the region. These findings demonstrate that density dependence and temporal autocorrelation influence reintroduced ungulate dynamics in arid systems. Management strategies that rely on short‐term counts risk misinterpreting recovery if past conditions continue to affect present growth. We recommend that conservation programmes incorporate multi‐year monitoring to detect delayed responses, anticipate density‐dependence slowdowns, and adapt interventions accordingly. Expanding consistent long‐term data collection across species and sites will be critical for improving the effectiveness of ungulate conservation in the Middle East.

## Graphical abstract

Using long‐term population data from Arabian oryx and sand gazelle populations across the Middle East, we found consistent evidence of negative density dependence, with annual growth rates declining as population size increased, alongside evidence of temporal lag effects in Arabian oryx. These results show that density‐dependent regulation is a key feature of ungulate dynamics in arid systems and highlight the need for long‐term monitoring to guide adaptive conservation management.

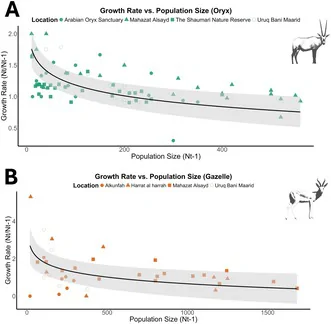

## 1 Introduction

Biodiversity loss is occurring at a rate that far exceeds historical baselines, positioning Earth in the midst of the sixth mass extinction driven by habitat loss, climate change, and overexploitation (Ceballos et al. 2015; Ellis and Ramankutty 2008). This persistent loss disrupts nature and diminishes essential ecosystem services such as pollination, water purification, and climate regulation (Cardinale et al. 2012; Gaston and Spicer 2004). To confront these declines, a range of conservation strategies have been implemented, including habitat protection, sustainable land‐use practices, and the restoration of populations (Foley et al. 2005; Mace 2014; Seddon et al. 2014).

Among these strategies, wildlife translocation, including reintroductions and reinforcements, has become a widely applied method to restore populations, re‐establish ecological interactions, and reduce extinction risk (Griffith et al. 1989; Fischer and Lindenmayer 2000; Ewen et al. 2012; Batson et al. 2015). However, the success of translocations is highly variable, depending on factors such as habitat suitability, species‐specific life history requirements, and prevailing environmental conditions (IUCN/SSC 2013). Socioecological conditions can be equally important, including social licence, support from local communities, poaching, and competition with livestock, as illustrated by concerns surrounding reintroduced wood bison ( *Bison bison athabascae* ) in Canada and by ungulate conservation programmes in Oman and Saudi Arabia (Spalton et al. 1999; Cunningham and Wacher 2009; Jung 2020). Alongside these socioecological constraints, reported failures highlight that ecological interactions and demographic feedbacks, such as competition and density‐related effects, can undermine recovery when they are overlooked in planning (Armstrong et al. 2005; Berger‐Tal et al. 2020). Effective reintroduction strategies must therefore account for the ecological feedback mechanisms that support the natural regulation of populations to prevent unintended outcomes that compromise long‐term persistence (Sarrazin and Barbault 1996; Hobbs et al. 2024).

These global lessons are especially important in regions like the Middle East, where conservation efforts since the 1970s have relied heavily on translocations to combat drastic ungulate declines through captive breeding and the establishment of protected areas (Spalton et al. 1999; Abuzinada 2003; Mésochina et al. 2003). While these interventions have supported the persistence or partial recovery of some populations, long‐term viability remains uncertain (Islam et al. 2011; Barichievy et al. 2018; Spalton et al. 1999; Seddon et al. 2014). Iconic species such as the sand gazelle (*Gazella marica*), Nubian ibex ( *Capra nubiana* ), and Arabian oryx ( *Oryx leucoryx* ), once widespread across the Arabian Peninsula, now persist mostly in small, human‐managed reserves (Thouless et al. 1991; Habibi 1986; Ripple et al. 2015; Pringle et al. 2023). The severity of these regional declines is further underscored by the extinction of the Saudi gazelle ( *Gazella saudiya* ), Syrian wild ass ( *Equus hemionus hemippus* ), Yemeni gazelle ( *Gazella bilkis* ), and bubal hartebeest ( *Alcelaphus buselaphus buselaphus* ) over recent decades (IUCN SSC Antelope Specialist Group 2016a, 2016b, 2017; Kaczensky et al. 2025). These mixed outcomes highlight the difficulty of maintaining viable populations in desert environments, where ecological conditions can strongly influence reintroduction success (Cunningham and Wacher 2009; Islam et al. 2010, 2011).

Arid ecosystems, which predominate in the Middle East, present additional challenges for conservation practice (Previtali et al. 2009). Unlike temperate regions, primary productivity in arid habitats is low and water availability fluctuates dramatically (Snyder and Tartowski 2006). Many desert species have evolved behavioural and physiological adaptations to cope with variable rainfall (Noy‐Meir 1973). However, their survival is increasingly threatened by habitat fragmentation, human–wildlife conflict, and intensifying climate variability (Vale and Brito 2015). These environmental pressures are compounded by socioecological constraints, including competition with domestic livestock and competing interests over grazing and access to protected areas (Cunningham and Wacher 2009). In such resource‐limited environments, simply increasing a species' population is not enough to ensure recovery, as ecological constraints like forage scarcity and competition can undermine long‐term conservation objectives (Read et al. 2021; Croft and Witte 2021). Moreover, the ecological processes regulating population growth are usually overlooked because small, fenced reserves restrict natural movements and supplementary feeding buffers populations from resource scarcity, limiting opportunities to observe population regulation directly (Islam et al. 2011; Cunningham and Wacher 2009).

Investigating population dynamics can provide important insights into species' demographic trends and persistence (Hixon et al. 2002; Allen et al. 2022; Harris et al. 2022). Key factors such as population size, reproductive rates, survival, and resource competition shape a species' viability (Dańko et al. 2018; Layton‐Matthews et al. 2019). At high densities, competition for limited resources (food, water, space) can suppress population growth rates (Sibly et al. 2005; Islam et al. 2011), while at very low population densities, species may suffer Allee effects, where difficulty finding mates or engaging in cooperative behaviours reduces survival or reproduction (Courchamp et al. 1999; Kramer et al. 2009). Recognising density‐dependent processes is important for wildlife conservation because they influence reintroduction outcomes, shape long‐term population dynamics, and affect extinction risk by stabilising demographic rates and buffering populations against environmental stress (Armstrong and Seddon 2008; Bretagnolle et al. 2008; Ferrer et al. 2014). While both climatic variation and competition modulate resource availability, the influence of density‐dependent responses (or negative feedbacks) on physiological status and reproduction can be more pronounced during periods of environmental stress (Bowyer et al. 2014). Because density‐dependent and density‐independent factors are now viewed as interacting (Sæther 1997), and the ability to detect density dependence is often a function of the climatic regime (Howell et al. 2002), it is essential to consider biotic regulation within resource‐limited systems (Xiao et al. 2019; Zhang et al. 2023).

The absence of systematic monitoring in the Middle East has left key ecological dynamics unexplored, particularly density‐dependent processes that are central to understanding reintroduction outcomes and species persistence in resource‐limited landscapes (Bowyer et al. 2014; Koons et al. 2015; Hobbs 2024). This gap reflects broader regional limitations in demographic data, as many ungulate species have fragmented or outdated population assessments (IUCN/SSC Antelope Specialist Group 2009; Mésochina et al. 2003). A search of the Living Planet Database in 2024 identified only nine terrestrial mammal populations from the region (LPI 2024). Similarly, our review of IUCN Red List assessments conducted in 2024 indicated that nearly half of the Least Concern species examined had unknown population trends, while most large desert ungulates were assessed as declining. While studies have documented separate population declines, no comprehensive analysis has assessed ungulate trajectories across the regional network of protected areas (Islam et al. 2011; Barichievy et al. 2018), leaving the prevalence and strength of empirical density‐dependent patterns largely undetected in this geographic context (Bonenfant et al. 2009).

To address this knowledge gap, we aimed to provide a data‐driven, comparative approach using two species, the Arabian oryx and sand gazelle (Figure 1). These taxa were identified through preliminary data exploration as suitable focal taxa because they combine long‐term monitoring coverage with distinct ecological traits. The Arabian oryx is a large‐bodied, slow‐reproducing grazer typically associated with open plains, whereas the sand gazelle is smaller, more mobile, and adapted to arid shrublands and patchy vegetation (Ostrowski and Williams 2006; Hetem et al. 2012). Their contrasting life histories and habitat preferences offer a valuable comparative framework for understanding how density‐dependent regulation operates across different ecological strategies in arid protected areas.

**FIGURE 1:**
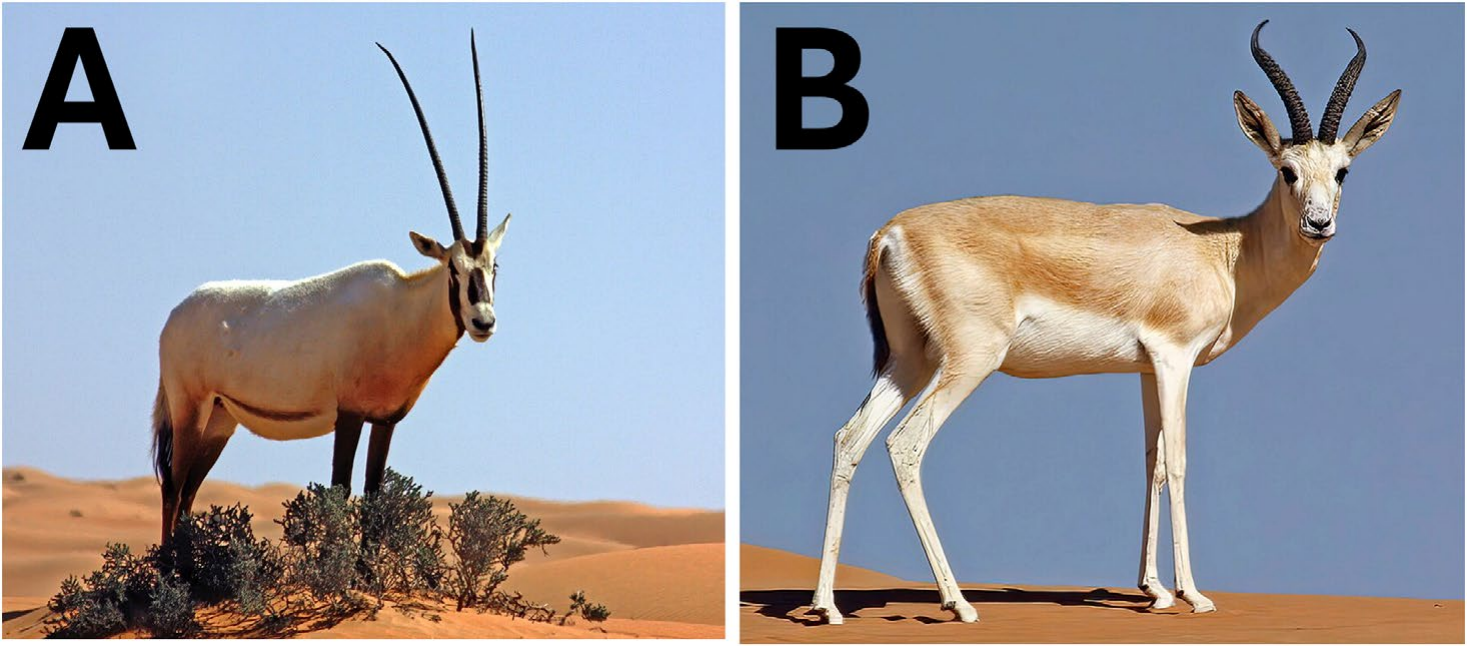
Focal ungulate species included in the population analyses. (A) Arabian oryx ( *Oryx leucoryx* ) and (B) sand gazelle (*Gazella marica*), photographed in the Dubai Desert Conservation Area, United Arab Emirates. Photographs by Charles J. Sharp via Wikimedia Commons licensed under CC BY‐SA 3.0 and CC BY SA 4.0, respectively.

Using linear mixed‐effects models (LMMs) across study sites, we assessed density‐dependent patterns, Allee effects, and temporal autocorrelation in the population trajectories. Our objectives were to: (i) determine the form and strength of density dependence in oryx and gazelle populations, (ii) test for any Allee effects at low densities, and (iii) consider the implications of these dynamics for conservation management in arid ecosystems. This evidence‐based analysis provides insight into population regulation in the two keystone desert herbivores and offers guidance for improving reintroduction success and long‐term population recovery in the region.

## 2 Methods

### 2.1 Data Extraction

The population series were identified through an initial literature screening conducted in February 2024 using Web of Science. Searches combined ungulate species' scientific and common names, including synonyms and former names, in English, Arabic, Hebrew and French, with the names and common abbreviations of Middle Eastern countries to identify published population records from the region. Population time series for Arabian oryx and sand gazelle were compiled from the published sources listed in Table 1. The final dataset comprised four Arabian oryx and four sand gazelle populations from protected areas in Jordan, Oman and Saudi Arabia. A series was retained when it represented a geographically defined population of a single species, contained repeated abundance observations across multiple years, and provided sufficient documentation of abundance values and monitoring methods to support interpretation of temporal change. Experimental datasets and populations maintained solely in captive‐breeding facilities were excluded. To reduce methodological heterogeneity, abundance records were standardised within each population by prioritising a consistent monitoring method across years. Where multiple comparable observations obtained using the same monitoring method were available within a given year, these were averaged to derive a single annual abundance value. Where observations from the preferred monitoring method were unavailable, the most comparable available estimate was retained. Remaining differences in monitoring and abundance‐estimation approaches are reported in Table 1.

**TABLE 1:** Population time series included in the analysis for Arabian oryx ( *Oryx leucoryx* ) and sand gazelle (*Gazella marica*) across six protected areas in Jordan, Oman, and Saudi Arabia. For each population, the table summarises the population history, monitoring method, data‐extraction procedure, and primary published source.

Species	Site	Years analysed	Population origin	Monitoring method	Data extraction	Primary data source
Arabian oryx ( *Oryx leucoryx* ) 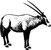	Shaumari Nature Reserve, Jordan	1978–2005 (28 annual observations)	Reintroduced; 11 initial animals imported during 1978–1979	Annual population totals reconstructed from reserve records of births, deaths, imports, exports and removals	Extracted from figure 2	Harding et al. (2007)
	Mahazat Alsayd, Saudi Arabia	1988–2009 (22 annual observations)	Reintroduced population; the published series begins in 1988, with a further 82 individuals released between 1990 and 2009	Annual population estimates based on accumulated births and deaths recorded by field workers, supplemented and cross‐checked by vehicle and aerial transect surveys	Extracted from figure 6	Islam et al. (2011)
	Uruq Bani Maarid, Saudi Arabia	1995–2010 (16 annual observations)	Reintroduced; releases began in 1995, with 174 oryx translocated by 2010	Intensive post‐release monitoring supplemented by vehicle and aerial surveys along four predefined transects; values represent population estimates	Extracted from figure 7	Islam et al. (2011)
	Arabian Oryx Sanctuary, Oman	1982–1999 (18 annual observations)	Reintroduced; 10 oryx were released in 1982 with subsequent releases bringing the total released to 40 by 1995	Direct individual counts during 1982–1990; Petersen mark–recapture estimates during 1991–1999	Extracted from figure 2a	Spalton et al. (1999)
Sand gazelle (*Gazella marica*) 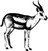	Mahazat Alsayd, Saudi Arabia	1991–2008 (18 annual observations)	Reintroduced; 164 sand gazelles were introduced during 1991–1994	Annual population estimates derived using distance sampling from regular transect surveys	Extracted from the numerical annual series reported	Islam et al. (2010)
	Alkhunfah Reserve, Saudi Arabia	1989–1993, 1995, 2001–2002 and 2005 (9 observations)	Remnant indigenous wild population	Initial vehicle count in 1989 followed by aerial surveys; retained values comprise direct counts and extrapolated or reported population estimates	Extracted from table 1	Cunningham and Wacher (2009)
	Harrat alharrah Reserve, Saudi Arabia	1989–1990, 1992–1996, 2002 and 2005 (9 observations)	Remnant indigenous wild population	Aerial surveys; retained values comprise direct counts and extrapolated or reported population estimates	Extracted from table 1	Cunningham and Wacher (2009)
	Uruq Bani Maarid, Saudi Arabia	1995, 1999–2000, 2002, 2005–2006 and 2008 (7 observations)	Reintroduced; 204 sand gazelles were introduced during 1995–1996	Predominantly vehicle surveys, with aerial survey data used where vehicle‐based estimates were unavailable; retained values comprise direct counts and extrapolated estimates	Extracted from table 1	Cunningham and Wacher (2009)

Across the included sources, abundance was quantified using several monitoring approaches. Arabian oryx records included reconstructed demographic totals, direct counts, mark–recapture estimates and transect‐informed population estimates (Harding et al. 2007; Spalton et al. 1999; Islam et al. 2011), whereas sand gazelle records included distance‐sampling estimates, direct counts and extrapolated survey estimates (Islam et al. 2010; Cunningham and Wacher 2009). Values were extracted from numerical series or published tables, while oryx values presented only graphically were digitised from the original figures. Source‐specific monitoring methods and extraction procedures are detailed in Table 1. Where monitoring years were non‐consecutive, missing years were not interpolated, and population change was calculated between successive available abundance observations. Comparable multi‐year abundance records were not available for other regional ungulates.

### 2.2 Study Areas and Population Context

The eight population series represented six protected areas in Jordan, Oman, and Saudi Arabia (Figure 2, Table 2). Arabian oryx populations were monitored at Shaumari Nature Reserve, the Arabian Oryx Sanctuary, Mahazat Alsayd, and Uruq Bani Maarid, all of which were reintroduced populations. Sand gazelle populations were monitored at Mahazat Alsayd, Uruq Bani Maarid, Alkunfah, and Harrat al harrah. The sand gazelle populations at Mahazat Alsayd and Uruq Bani Maarid were reintroduced, whereas those at Alkhunfah and Harrat al harrah represented remnant indigenous populations. The protected areas differed in reserve size, fencing, rainfall, access to surrounding rangelands, management interventions, and documented socioecological pressures, which are summarised in Table 2.

**FIGURE 2:**
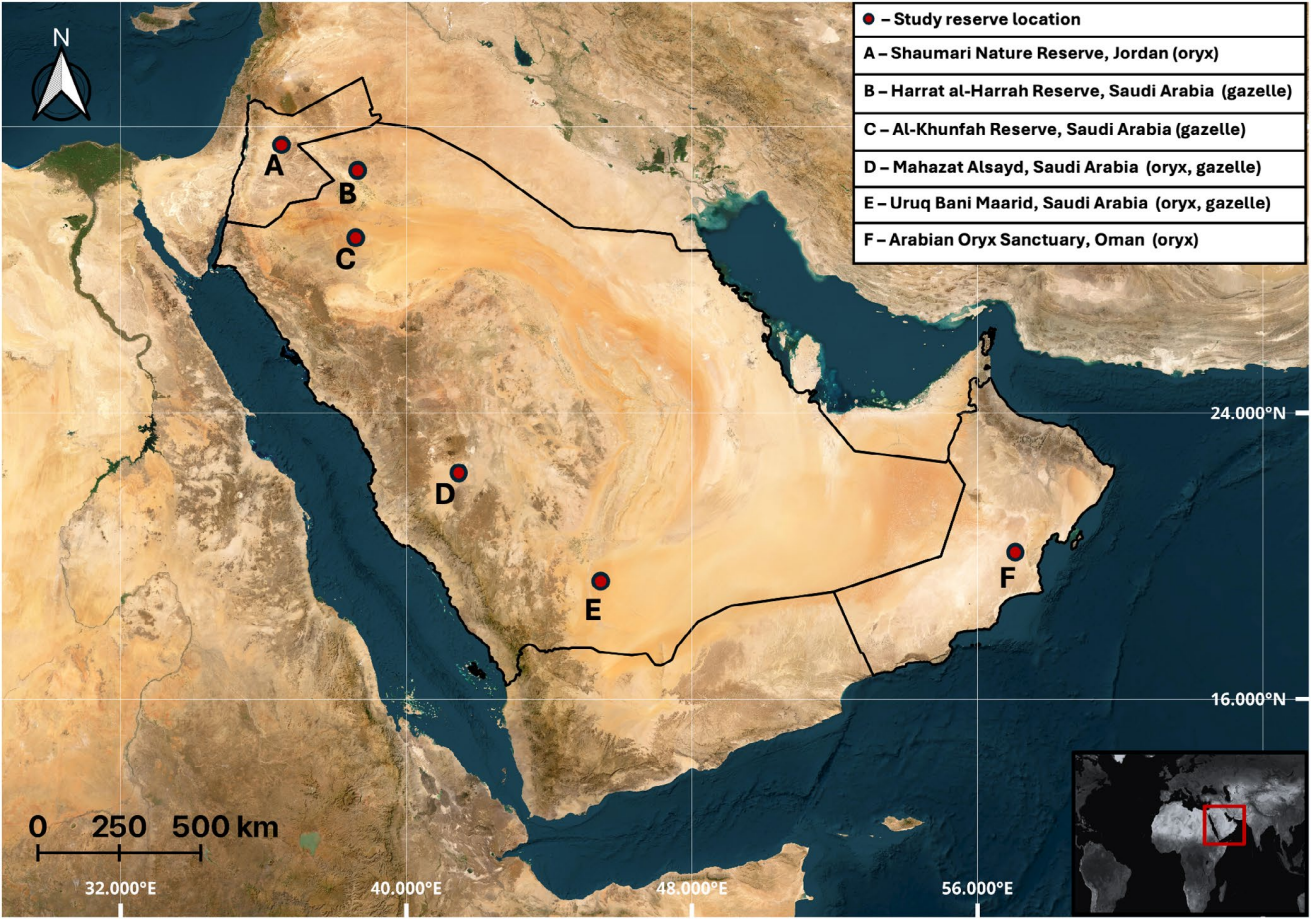
Map of the Middle East showing the locations of study reserves for the Arabian oryx ( *Oryx leucoryx* ) and sand gazelle (*Gazella marica*) over a satellite‐imagery basemap.

**TABLE 2:** Ecological, management and socioecological context of the six protected areas represented in the Arabian oryx ( *Oryx leucoryx* ) and sand gazelle (*Gazella marica*) analyses.

Site	Study population	Habitat and rainfall	Reserve area (km^2^)	Management and socioecological context	Primary source
Shaumari Nature Reserve, Jordan	Arabian oryx	Arid hammada with sparse grasses and forbs in wadis; mean annual rainfall 62.2 mm	342 km^2^ reserve, a 22 km^2^ section was completely fenced in 1984 to contain wildlife and exclude livestock	Water and shade were provided through a well and planted trees. Livestock influx associated with the 1990–1991 Gulf War caused severe overgrazing in surrounding rangelands and prevented wider release	Harding et al. (2007)
Arabian Oryx Sanctuary, Oman	Arabian oryx	Hyper‐arid flat to undulating plateau with sandy depressions; mean annual rainfall 39 mm, no permanent natural water	Approximately 25,000 km^2^; free‐ranging landscape. Oryx occupied more than 16,000 km^2^ by 1995	Supplementary food and water ceased in 1986, and local Bedouin were employed as rangers. Illegal capture from 1996 caused a severe population decline and prompted removal of animals to a protected captive herd. The sanctuary was later reduced by 90% and removed from the UNESCO World Heritage List in 2007	Spalton et al. (1999); UNESCO World Heritage Centre (2007)
Mahazat Alsayd Protected Area, Saudi Arabia	Arabian oryx and sand gazelle	Arid sandy and gravel plain; highly variable annual rainfall, approximately 50–100 mm	2244 km^2^; fully fenced by 1989 to exclude livestock and poachers. The fence also restricts long‐distance movement in response to drought and rainfall	Livestock exclusion allowed vegetation recovery. During drought, restricted movement and declining forage availability contributed to substantial mortality. Emergency food and water provision and population removals or translocations were subsequently used as management responses	Islam et al. (2010, 2011); Cunningham and Wacher (2009)
Uruq Bani Maarid Protected Area, Saudi Arabia	Arabian oryx and sand gazelle	Western Rub al‐Khali dunes, interdune corridors, gravel plains and limestone escarpment; mean annual rainfall < 47 mm; no permanent water sources	12,658 km^2^; unfenced, comprising a 2400 km^2^ core reserve, a managed‐grazing zone and a wider controlled‐hunting zone	The unfenced setting permits movement beyond the protected‐area boundary. Poaching affected the oryx population during the study period, while competition with livestock was identified as a potential management concern	Islam et al. (2011); Cunningham and Wacher (2009)
Alkhunfah Reserve, Saudi Arabia	Sand gazelle	Sand and gravel plain with sandstone hills along the western Nafud margin; annual rainfall 100–200 mm	20,450 km^2^; approximately 2000 km^2^ was actively protected by ranger stations and an earth barrier restricting vehicle access	Uncontrolled hunting and competition with domestic livestock, particularly camels, were identified as major pressures on the population. Farming along the southern boundary added further pressure	Cunningham and Wacher (2009)
Harrat al harrah Reserve, Saudi Arabia	Sand gazelle	Undulating basalt plains with volcanic ash, aeolian sands and salt flats; annual rainfall 50–100 mm	13,775 km^2^; subsequently reduced to approximately 7990 km^2^, with an earth barrier and pipeline forming partial barriers	Reduction of the protected area was associated with local opposition concerning grazing and access rights. Uncontrolled hunting and competition with domestic livestock were identified as major pressures on the population	Cunningham and Wacher (2009)

### 2.3 Analysis

To analyse the relationship between population size and growth, we calculated proportional population growth between successive available observations for each time series. Growth was defined as (*N*_*t*_/*N*_*t*–1_), where *N*_*t*_ is the current abundance observation and *N*_*t−*1_ represents the preceding available abundance observation within each population series, following Royama (1992) and Fowler (1987). For annually monitored series, this corresponds to annual population growth; where monitoring years were non‐consecutive, the ratio represents population change across the interval between successive surveys. We first performed exploratory data visualisation (Figure 3). Plotting growth rates against previous‐year population size showed a negative relationship for both species (Figure 4). We explored several candidate functional forms for the population size–growth relationship, including linear, inverse, quadratic, and logarithmic forms. Model selection was based on the Akaike information criterion (AIC), which balances goodness‐of‐fit and model complexity. It indicated that a log‐linear relationship provided the best fit for both species, with no substantial improvement from more complex nonlinear forms. We therefore proceeded with the log‐linear models where growth rate declines with increasing prior‐year population size.

**FIGURE 3:**
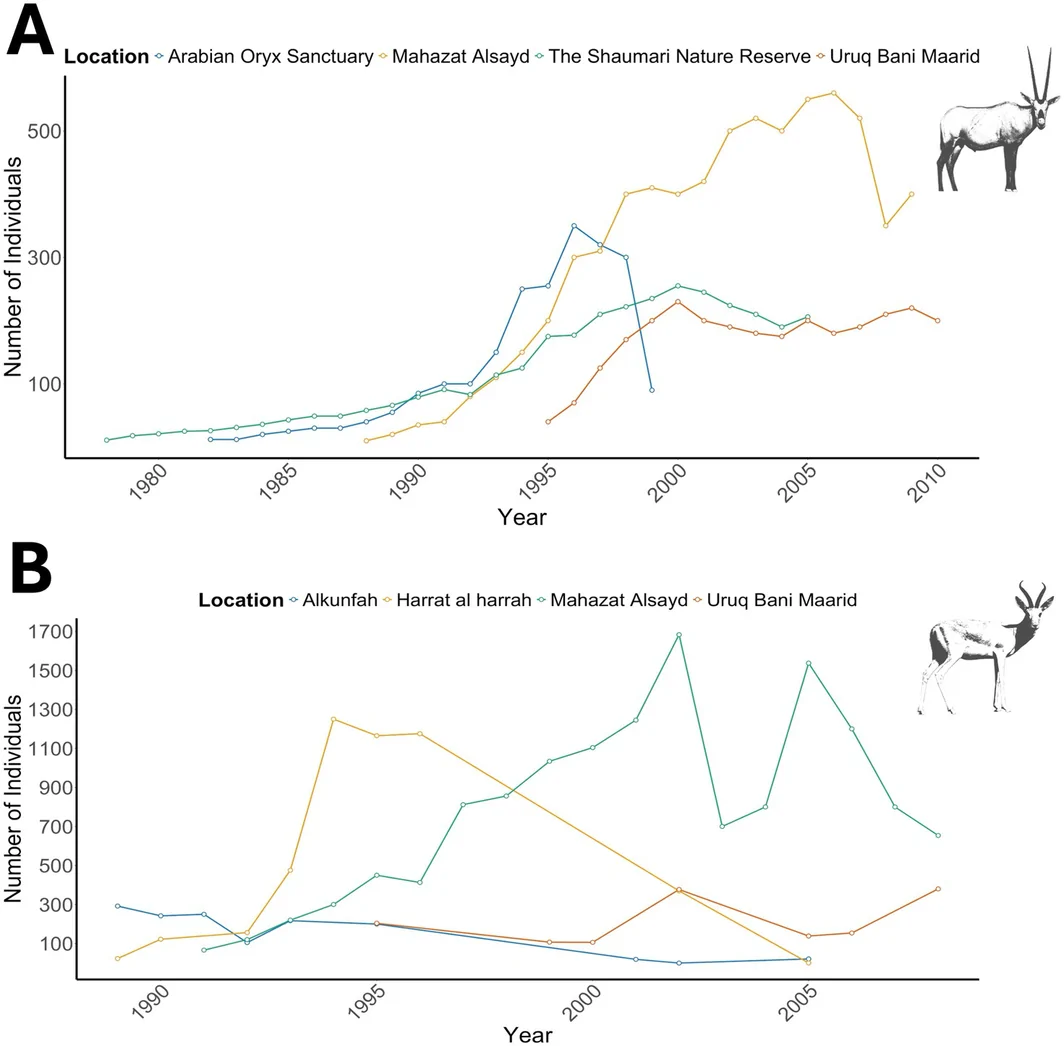
Population trajectories of Arabian oryx (A) and sand gazelle (B) across study populations. Initial reintroduction years were: Shaumari, 1978; Arabian Oryx Sanctuary, 1982; Mahazat Alsayd, 1988 (oryx) and 1991 (gazelle); and Uruq Bani Maarid, 1995 (oryx and gazelle). Alkhunfah and Harrat alharrah represent remnant sand gazelle populations. See Table 1 for details.

**FIGURE 4:**
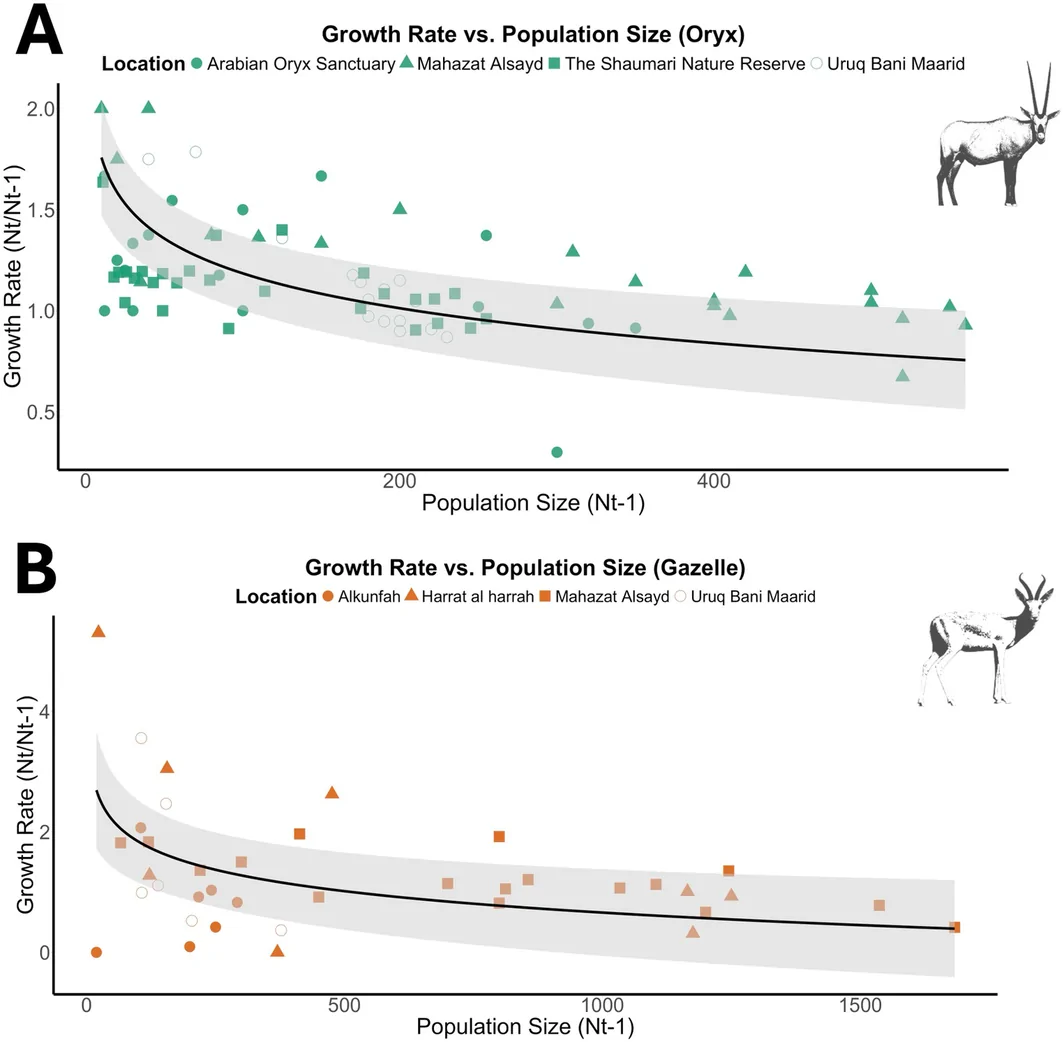
Density‐dependent population growth in Arabian oryx and sand gazelle across monitored reserves. (A) Scatterplot of annual growth rate versus prior‐year population size for Arabian oryx, with each point representing an observation from a specific location. The solid black line indicates the fitted relationship from the AR(2) mixed‐effects model, and the shaded area represents the 95% confidence interval. (B) Similar plot for sand gazelle, with the fitted line derived from a random intercept model. Both species exhibit a negative relationship between population size and growth rate, suggesting consistent density‐dependent regulation across sites. The oryx data show tighter clustering around the trend line, while the gazelle data display greater variability.

Given that the data for each species encompassed multiple time series labelled with unique locations, we employed linear mixed‐effects models (LMMs) to account for the hierarchical structure of the data (Bolker et al. 2009; Oberpriller et al. 2022). Location was treated as a random effect, allowing for site‐specific intercepts, and potentially slopes, to capture unmeasured differences between reserves (e.g., habitat quality, management regime). Population size (*N*_*t*–1_) was included as a fixed effect to estimate the overall influence of crowding on growth rate. We fitted two main mixed model structures for each species: (i) a random intercept‐only model, where baseline growth rates varied across locations, and (ii) a random intercept and slope model, allowing both baseline growth rates and the effect of population size on growth to vary by location. We used AIC comparisons to evaluate whether the random‐slope model provided a better fit than the simpler random intercept‐only model.

We also evaluated temporal autocorrelation in the population trajectories to determine whether multi‐year lag effects influenced population growth. During model diagnostics, we assessed whether year‐to‐year growth fluctuations were independent by examining autocorrelation in the model residuals. We used autocorrelation function (ACF) plots to identify any patterns of temporal dependence in the time series. For Arabian oryx time series diagnostics, the ACF revealed several correlations at specific lags, which prompted us to incorporate temporal structure into the model. We implemented autoregressive error terms by first fitting an AR(1) model that included a 1‐year lag, followed by an AR(2) model that accounted for up to 2‐year lags. For sand gazelles, the time series were shorter and contained gaps. Therefore, we did not attempt to fit AR terms for gazelles, as the data were insufficient to reliably estimate autocorrelation. All models were fitted in R 4.2.2 using lme4 and nlme packages (Bates et al. 2015). Model assumptions (normality and homoscedasticity of residuals) were checked via residual plots.

## 3 Results

The analysis included 84 Arabian oryx and 43 sand gazelle abundance records across four populations of each species. Arabian oryx populations were represented by Shaumari Nature Reserve, the Arabian Oryx Sanctuary, Mahazat Alsayd and Uruq Bani Maarid, while sand gazelle populations were represented by Mahazat Alsayd, Uruq Bani Maarid, Alkhunfah and Harrat alharrah. Summary statistics indicate that oryx counts ranged roughly from 10 to 560 individuals, whereas gazelle counts spanned 19 to 1683 individuals. Annual growth rates (*N*_*t*_/*N*_*t−*1_) averaged 1.17 for oryx and 1.32 for gazelles, with substantially higher variance in gazelles. This variability reflects differing population trajectories. Most oryx populations increased rapidly after reintroduction before levelling off or fluctuating, with Mahazat Alsayd reaching the highest abundance, whereas gazelle trajectories were more variable, including pronounced fluctuations at Mahazat Alsayd and a marked decline at Harrat alharrah. Notably, none of the monitored populations exhibited unchecked exponential growth, even in the early years following reintroduction (Figure 3).

### 3.1 Density Dependence

The models indicate a negative density dependence trend in both species as shown in Table 3. The best‐fit models for both oryx and gazelle indicated a decline in annual growth rate with increasing population size. In the Arabian oryx, our model yielded a significant negative relationship between annual growth rate and prior‐year population size. A log‐linear fixed‐effects model provided a better fit based on AIC (1.79), with a slope of (−0.14 ± 0.02; *p* < 0.001). Similarly, sand gazelles showed negative density dependence with the log‐linear model also performing better than the linear version (AIC 109.16 vs. 111.90), with an estimated slope of (−0.39 ± 0.14; *p* = 0.009). Additionally, there was no evidence of Allee effects in the analysed populations. No drop in growth rate was observed at very low population sizes, and the smallest population sizes corresponded to some of the highest growth rates (Figure 4).

**TABLE 3:** Summary of candidate models relating population growth rates to population size (P Size) for Arabian oryx ( *Oryx leucoryx* ) and sand gazelle (*Gazella marica*) across multiple protected areas. Model types include fixed‐effects (linear, quadratic, log‐linear), mixed‐effects with random intercepts (site‐level variation), and autoregressive models (AR(1) and AR(2)) for temporal autocorrelation. Estimates include model degrees of freedom (df), AIC, parameter coefficients (*β* ± SE), associated *p*‐values, and model fit (adjusted *R* ^2^ for fixed models; conditional/marginal *R* ^2^ for mixed models). AR coefficients (Φ₁, Φ₂) indicate lag structure; random effects standard deviations (SD) reflect among‐site variability. The best‐fit model for oryx included both random intercepts and AR(2) terms; for gazelles, the random‐intercept model is reported because the random‐slope model was singular. Models were selected based on AIC and diagnostic improvement.

Species	Model	df	AIC	*β* ± SE	*p*	*R* ^2^ (adj.) (cond./marg.)	Random effects
Arabian Oryx 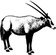	Linear	3	6.11	Intercept: 1.17 ± 0.03	< 0.001	0.229 (adj.)	NA
				P Size: −0.0009 ± 0.0002	< 0.001		
	Quadratic	4	3.37	Intercept: 1.12 ± 0.04	< 0.001	0.264 (adj.)	NA
				P Size: −0.0013 ± 0.0002	< 0.001		
				P Size^2^: 0.000002 ± 0.000001	0.033		
	Log‐linear	3	1.79	Intercept: 1.81 ± 0.12	< 0.001	0.270 (adj.)	NA
				Log(P Size): −0.14 ± 0.02	< 0.001		
	Random intercept	4	7.78	Intercept: 1.96 ± 0.13	< 0.001	0.469/0.351	Intercept SD: 0.1
				Log(P Size): −0.17 ± 0.02	< 0.001		
	Random intercept + AR(1)	5	5.34	Intercept: 2.04 ± 0.16	< 0.001	0.502/0.380	Intercept SD: 0.11 AR(1) Φ = 0.30
				Log(P Size): −0.18 ± 0.03	< 0.001		
	**Random intercept + AR(2)**	**6**	**−0.76**	**Intercept: 2.33 ± 0.24**	**< 0.001**	**0.542/0.454**	**Intercept SD: 0.12** **AR(2) Φ₁ = 0.30** **Φ₂ = 0.42**
				**Log(P Size): −0.25 ± 0.05**	< 0.001		
	Random slope	6	2.69	Intercept: 2.28 ± 0.49	< 0.001	0.735/0.393	Intercept SD: 0.92; slope SD: 0.18
				Log(P Size): −0.22 ± 0.09	0.018		
Sand Gazelle 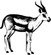	Linear	3	111.90	Intercept: 1.31 ± 0.16	< 0.001	0.088 (adj.)	NA
				P Size: −0.0007 ± 0.0004	0.039		
	Quadratic	4	113.32	Intercept: 1.18 ± 0.26	< 0.001	0.076 (adj.)	NA
				P Size: −0.00096 ± 0.00048	0.050		
				P Size^2^: 0.0000006 ± 0.0000009	0.471		
	Log‐linear	3	109.16	Intercept: 3.58 ± 0.83	< 0.001	0.152 (adj.)	NA
				Log(P Size): −0.39 ± 0.14	0.009		
	**Random intercept**	**4**	**112.57**	**Intercept: 4.21 ± 0.87**	**< 0.001**	**0.433/0.240**	**Intercept SD: 0.52**
				**Log(P Size): −0.51 ± 0.15**	**0.0013**		
	Random slope^**a**^	6	109.91	Intercept: 3.62 ± 1.61	NA	NA	Intercept SD: 2.91; slope SD: 0.44 (corr = −1.00)
				Log(P Size): −0.38 ± 0.25			

*Note:* Bold values indicate the final model retained for inference for each species. For Arabian oryx, the random‐intercept model with an AR(2) error structure was selected; for sand gazelle, the random‐intercept model was selected, as the random‐slope model was singular and was therefore not used for inference.

^a^ Singular/unreliable and therefore not used for inference.

We also examined whether the density‐dependence relationship varied among sites. For Arabian oryx, allowing the density slope to vary among sites reduced AIC relative to the random‐intercept model. For sand gazelles, the random‐slope model was singular and was therefore not used for inference. To describe site‐level differences in baseline growth, we report the random‐intercept models here, while temporal structure in the Arabian oryx model is evaluated separately below. In the Arabian oryx random‐intercept model, the intercept was 1.96 ± 0.13 (*p* < 0.001) and the log(Population Size) slope was −0.17 ± 0.02 (*p* < 0.001), with a random‐intercept standard deviation of 0.10. For sand gazelles, the random‐intercept model yielded an intercept of 4.21 ± 0.87 (*p* < 0.001) and a log(Population Size) slope of −0.51 ± 0.15 (*p* = 0.0013), with a random‐intercept standard deviation of 0.52. Across sites, the fitted relationships remained negative, while the random intercepts captured differences in baseline growth among locations (Figures 4 and 5B,C).

**FIGURE 5:**
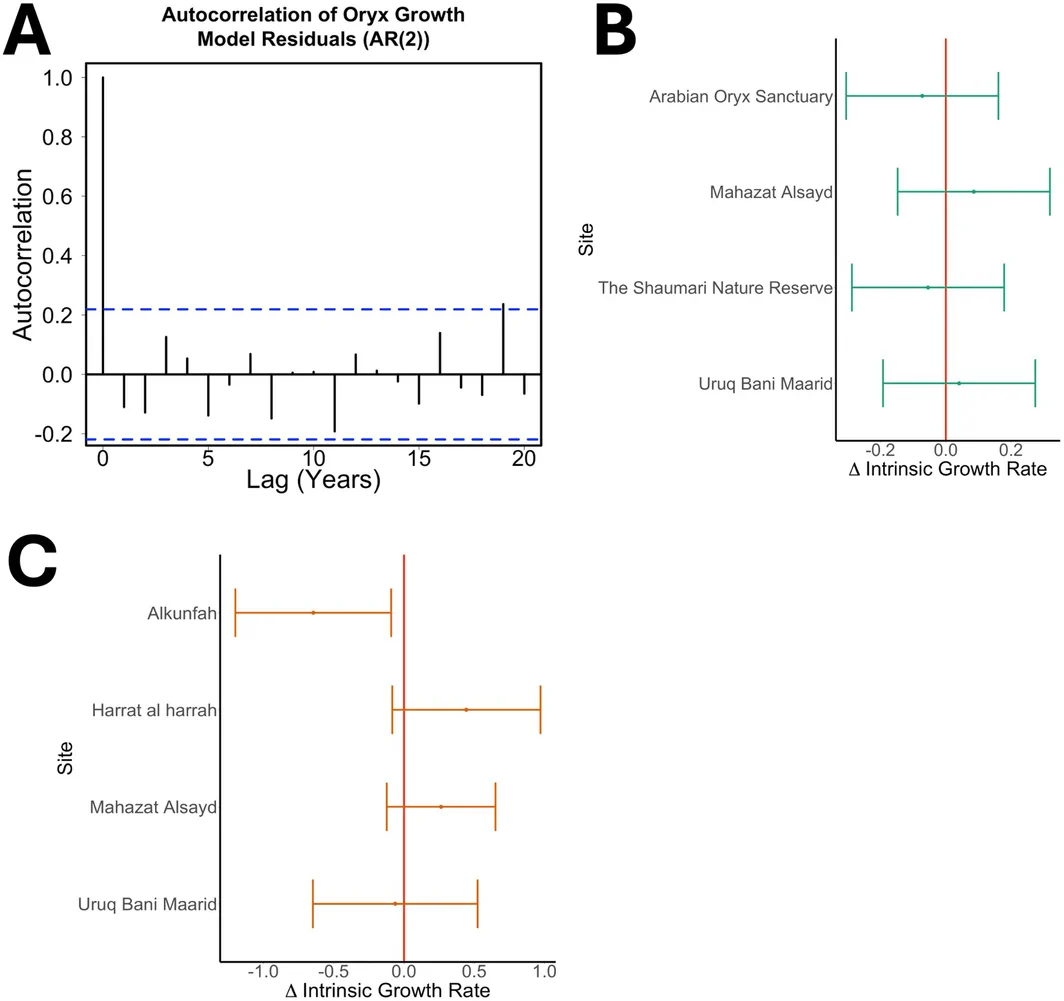
(A) Autocorrelation function (ACF) of Arabian oryx AR(2) model residuals. (B, C) Site‐level deviations in intrinsic growth rates from mixed‐effects models with random intercepts for (B) Arabian oryx and (C) sand gazelles. Points represent each site's deviation from the species‐average intercept (Δ intrinsic growth rate), with 95% CI. Positive values indicate sites with higher‐than‐average baseline growth, and negative values indicate lower‐than‐average growth potential.

### 3.2 Temporal Autocorrelation

The long time series for Arabian oryx revealed an additional pattern of temporal autocorrelation in growth rates. We examined the autocorrelation function (ACF) of our data to see if year‐to‐year growth fluctuations were independent, by incorporating an autoregressive error structure into the oryx model. An AR(1) term (1‐year lag) with coefficient Φ ≈0.30 improved the model (AIC ≈5.34), indicating that including a memory of the previous year's conditions provides a better fit. Furthermore, an AR(2) model (including up to 2‐year lags) improved fit even more (AIC ≈−0.76) with significant coefficients at lag‐1 (~0.30) and lag‐2 (~0.42). Therefore, the preferred model for oryx indicates that population growth in a given year was influenced not only by the current population size but also by population conditions in the prior 1 to 2 years.

Diagnostic checks of the final model residuals revealed that while the AR(2) structure effectively accounted for short‐term temporal autocorrelation, a significant lag remained at year 19 in the ACF plot (Figure 5A). Visual inspection of residuals against time indicated a long‐term cyclic pattern that was not fully captured by the density‐dependent or autoregressive terms. Importantly, the estimated density effect remained negative after incorporating AR(1) and AR(2) temporal structures, indicating that accounting for short‐term temporal dependence did not remove the negative population‐size–growth relationship as seen in Table 3. For sand gazelles, we did not fit autoregressive models due to the shorter and more fragmented time series, which contained missing years.

## 4 Discussion

In this study, we set out to evaluate how threatened ungulate populations in arid Middle Eastern protected areas are regulated over time, with a particular focus on identifying density‐dependent patterns across species and sites. Across the analysed Arabian oryx and sand gazelle populations, growth rates declined as population size increased. This negative population‐size–growth relationship is consistent with fundamental ecological theory and empirical research on large herbivores (Bowyer et al. 2014). Importantly, this relationship was present across populations occurring under contrasting management and ecological settings, including fenced and unfenced free‐ranging reserves. The consistency of the negative relationship across these different settings suggests that it was not restricted to populations in small, fenced reserves where movement and resource availability were constrained. Density‐dependent regulation is a well‐documented pattern in ungulate populations, where limited resources or space can lead to increased competition and lowered fecundity or survival as carrying capacity is approached (Gaillard et al. 2000; Bowyer et al. 2014; Bonenfant et al. 2009; Caughley 1977).

Within the analysed series, we found no evidence of positive density dependence at low abundance. The absence of a detected Allee effect should not, however, be interpreted as evidence that small populations are generally free from demographic risk. Rather, low abundance was not associated with a detectable reduction in growth in these time series, despite the potential for small populations to experience constraints such as mate limitation or social disruption (Courchamp et al. 1999; Berec et al. 2007). Overall, our findings suggest that negative density‐dependent feedback was the dominant pattern, with no detectable evidence of low‐number effects (Bowyer et al. 2014; Sinclair and Byrom 2006). Site‐level differences in baseline population growth were accommodated through the random intercept, while allowing the density‐dependence relationship itself to vary among reserves did not provide the preferred overall model structure. Within the limits of our time series, this supports a broadly consistent negative population‐size–growth relationship across the analysed reserves, despite differences in their underlying ecological and management contexts (Harrison et al. 2018).

The Arabian oryx showed a clear negative relationship between population size and growth across all sites, although the ecological conditions through which density dependence was expressed differed among reserves (Table 3, Figure 4A). This result is in line with other reintroduction case studies and population models for large ungulates, which demonstrate that once population size nears the environmental carrying capacity, growth decelerates and can even cease (Owen‐Smith 2006; Traill et al. 2007; Fryxell et al. 2010; Bowyer et al. 2014). If unmanaged, such density‐driven slowdowns can impede a population's progress toward long‐term viability. In extreme cases, an overshoot of carrying capacity followed by resource depletion may trigger population crashes. For example, in the fenced Mahazat Alsayd reserve in Saudi Arabia, the Arabian oryx population irrupted to hundreds of individuals within a decade of reintroduction, only to suffer a catastrophic die‐off during a drought when forage became scarce (Islam et al. 2011).

Similarly, sand gazelle populations demonstrated significant negative density dependence in their growth rates across all protected areas. Despite the gazelle's smaller body size and potentially higher intrinsic growth rate, even these herds experienced reduced growth rates at elevated densities (Table 3, Figure 4B). In fenced reserves in arid environments, food and water resources are finite, and as gazelle numbers increase, forage availability per capita drops, leading to nutritional stress and greater mortality (Blank et al. 2012; Islam et al. 2010; Ripple et al. 2015). This was particularly evident at Mahazat Alsayd, where rapid population growth was followed by substantial mortality during periods of drought and reduced forage availability, with juveniles especially susceptible to food shortage (Islam et al. 2010, 2011). However, the conditions surrounding the gazelle trajectories differed among reserves. The remnant populations at Alkhunfah and Harrat alharrah were also exposed to uncontrolled hunting and competition with domestic livestock, which contributed to their longer‐term declines (Cunningham and Wacher 2009). Although density dependence was present across the gazelle populations, the ecological and socioecological pressures affecting them varied among reserves.

In addition to density feedback, the Arabian oryx exhibited a distinct pattern of temporal autocorrelation in its population growth. In biological terms, the pattern aligns with the oryx's life history. Arabian oryx are relatively long‐lived, slow‐reproducing ungulates, with individuals capable of approaching 20 years of age and with overlapping generations (Ochoa et al. 2016). Species with overlapping generations can exhibit population momentum, where the effects of favourable or unfavourable conditions carry over for multiple years (Hixon et al. 2002). These lagged responses suggest that oryx growth trajectories may reflect both demographic inertia and unmeasured environmental drivers such as rainfall variability (Treydte et al. 2001). Incorporating temporal correlation into our models improved confidence in the population size effect, which remained robust across all variants. The remaining autocorrelation at lag 19 may indicate delayed feedback cycles in oryx population dynamics (Figure 5A).

These patterns are consistent with long‐term demographic or environmental influences that emerge over multiple generations (Royama 1992). Recognising the potential for delayed responses is important for managers working with long‐lived species. Although we could not assess autocorrelation in sand gazelles, their life history characteristics suggest that similar lagged demographic responses may occur, but the possibility remains untested due to data limitations. The relatively small number of populations available for each species warrants caution when interpreting among‐site variation. However, simulation studies indicate that population‐level fixed‐effect estimates from random‐intercept mixed models can remain reliable with few grouping levels, although estimates of random‐effect variation are less precise (Oberpriller et al. 2022). Interpretation of the gazelle results also requires some caution because, despite prioritising a consistent survey platform within each population, the historical series retained both direct counts and extrapolated estimates and varied in survey coverage and observers among years (Cunningham and Wacher 2009).

Beyond our two focal species, data limitations prevented robust assessments of density dependence in other threatened ungulates of the Arabian Peninsula. We compiled records in Table 4 for species such as the mountain gazelle ( *Gazella gazella* ), Arabian gazelle ( *Gazella arabica* ), dorcas gazelle ( *Gazella dorcas* ), and Nubian ibex, but long‐term, reliable population data for these taxa were scarce or fragmentary. In many cases, available numbers were outdated, sporadic, or derived from anecdotal reports rather than systematic monitoring, making it difficult to discern any density‐dependent trends (Collen et al. 2009; Cazalis et al. 2023). The available evidence may also be affected by publication and outcome‐reporting bias, as monitoring outcomes are not necessarily equally represented in the published literature (Marks‐Anglin et al. 2021). It is possible that similar negative density dependence operates in these species as well, since other ungulate populations worldwide commonly show density‐linked changes in survival and reproduction (Bonenfant et al. 2009; Eberhardt 2002; Milner et al. 2007; Bowyer et al. 2014). However, many of these populations remain well below carrying capacity due to external pressures, so population regulation processes may be minimal or masked under current conditions (Brito et al. 2018; Ogutu et al. 2016). Decades of external pressures, such as overhunting and habitat degradation, have kept some wild gazelle herds so small that they have yet to approach densities where resource competition becomes a dominant factor (Thouless et al. 1991). Instead, poaching, encroachment by livestock, and periodic drought can play a more immediate role in limiting these populations (Magin and Greth 1994; Ogutu et al. 2016; Daskin and Pringle 2018).

**TABLE 4:** Regional ungulate species considered but not included in the final population‐dynamics analysis owing to insufficient long‐term abundance data. IUCN Red List status and population trends are reported for each species according to the IUCN Red List (IUCN 2026).

Common name	Scientific name	IUCN Red List status	Population trend
Mountain gazelle	*Gazella gazella*	Endangered (EN)	Decreasing
Nubian ibex	*Capra nubiana*	Vulnerable (VU)	Decreasing
Arabian gazelle	*Gazella arabica*	Vulnerable (VU)	Decreasing
Dorcas gazelle	*Gazella dorcas*	Vulnerable (VU)	Decreasing
Arabian tahr	*Arabitragus jayakari*	Endangered (EN)	Decreasing
Persian fallow deer	*Dama mesopotamica*	Endangered (EN)	Increasing
Asiatic wild ass	*Equus hemionus*	Near Threatened (NT)	Stable

Indeed, in stressed or recovering populations, external mortality factors can effectively overshadow any density‐dependent regulation that might otherwise occur. This phenomenon has been observed in other regions as well, for example in war‐torn regions or those under intense anthropogenic pressure, where large mammals often decline due to human impacts long before natural density feedback becomes relevant (Craigie et al. 2010; Daskin and Pringle 2018; Trinkel et al. 2017). Such extrinsic threats culminated when UNESCO deleted Oman's Arabian Oryx Sanctuary from the World Heritage List in 2007, after the protected area was reduced by 90% and the oryx population declined from approximately 450 individuals in 1996 to 65, largely as a result of poaching and habitat degradation (UNESCO World Heritage Centre 2007). Together, these cases demonstrate that density‐dependent regulation can be fragile in arid systems and can be masked by either climatic extremes or extrinsic human pressures.

Our study, therefore, underscores not only the importance of density‐dependent processes but also the critical need to mitigate external threats and improve data coverage for ungulate species in the Middle East. Without better information on population trajectories, especially for species that are currently rare or fragmented, it remains challenging to determine whether their dynamics are driven more by density factors or by ongoing anthropogenic pressures (Alatawi 2022; Ogutu et al. 2016). Despite these limitations, the consistent density‐dependent patterns observed in two ecologically distinct species suggest that negative density dependence can be important in arid ungulate populations. The convergence between Arabian oryx and sand gazelle indicates that this pattern is not restricted to a single taxon, although comparable long‐term data from additional species are needed to determine how broadly it applies across desert ungulates (Lerp et al. 2016; Fadakar et al. 2021; Bowyer et al. 2014). Similarly, density‐dependent relationships have been reported in other reintroduced herbivores, from African antelope to Asian deer, reinforcing the concept that carrying capacity constraints and competition are fundamental aspects of herbivore population recovery (Hobbs 2024; Bonenfant et al. 2009; Bowyer et al. 2014).

In light of these findings, we highlight several critical directions for conservation management and monitoring of reintroduced ungulates in arid Middle Eastern ecosystems. Long‐term, consistent population monitoring emerges as an important need, given that population dynamics in deserts can be punctuated by episodic events (such as droughts and disease outbreaks) as well as slow, gradual trends. In practice, wildlife monitoring across this region remains limited. Arid drylands are among the world's least‐studied biomes, resulting in fragmented data and knowledge gaps (Durant et al. 2012; Brito et al. 2014; Cazalis et al. 2023). Such programmes would feed directly into conservation strategies by providing the feedback needed for adaptive management (Legg and Nagy 2006). An adaptive monitoring framework, where management actions are adjusted in response to regular population assessments, is especially suited to these reserves (Nichols and Williams 2006).

In addition to abundance counts, incorporating demographic indicators such as age structure, fecundity, and survival can reveal early warning signs of stress before total population decline is apparent (Eberhardt 2002; Gaillard et al. 2000). These parameters help diagnose whether density dependence arises through reduced juvenile recruitment, lowered adult survival, or suppressed reproduction, allowing managers to tailor interventions more precisely (Bonenfant et al. 2009). By linking monitoring data to management decisions in this way, conservation practitioners create a responsive, learning‐based system that can better ensure population stability (Legg and Nagy 2006). This approach aligns with best‐practice guidelines calling for iterative management informed by outcomes (IUCN/SSC 2013; Armstrong and Seddon 2008).

Our findings also emphasise the importance of an ecosystem‐based approach, looking beyond single‐species dynamics. Density dependence in these ungulates likely interacts with other ecological factors, and understanding these interactions can improve management outcomes (Hopcraft et al. 2010). Climatic variability, particularly multi‐year droughts, can intensify density‐dependent mortality when resource availability is sharply reduced. Even populations at moderate densities may suffer severe losses when forage and water become limiting. In previous cases, low rainfall has coincided with starvation and elevated mortality in reintroduced ungulates, showing that carrying capacity can contract sharply with rainfall (Islam et al. 2010). These events highlight that carrying capacity in arid landscapes varies with rainfall and vegetation productivity. We did not include climatic predictors (e.g., precipitation) because our focus was on density feedback. Future studies should examine how rainfall variability interacts with population regulation. Future management could integrate habitat and climatic information when assessing drought risk. Similarly, predator reintroductions, such as Arabian wolves ( *Canis lupus arabs* ) or Arabian leopards ( *Panthera pardus nimr* ) in some reserves, could modulate prey crowding and contribute to improved ecosystem balance. Investigating these dynamics through experimental or comparative studies would provide deeper insight into managing whole ecosystems rather than focusing solely on single species (Ripple et al. 2015; Hayward et al. 2007; Jachowski et al. 2020).

In summary, we show that density‐dependent regulation is a consistent process in protected populations of Arabian oryx and sand gazelle, with clear implications for conservation management. Recognising this pattern allows managers to anticipate natural slowdowns in growth and adjust strategies accordingly. Many protected areas in the Middle East are fenced and isolated, and function as critical refuges for these formerly extirpated species. Management in such systems must therefore be especially responsive to changes in population growth and habitat conditions (IUCN/SSC 2013). Management strategies should also distinguish between fenced and unfenced reserves, as closed systems may experience stronger density‐dependent effects and limited dispersal. In such cases, translocations and population augmentation must be carefully coordinated to prevent overstocking or genetic bottlenecks. Where possible, establishing corridors or facilitating genetic rescue may enhance long‐term viability, particularly for small or isolated populations (Frankham 2015). We recommend that managers use density‐feedback signals to guide decisions about when to augment, reduce, or connect populations. Where growth slows or signs of resource limitation emerge, managers may consider reducing density through translocation to suitable sites or, where appropriate, through controlled off‐take (Treydte et al. 2001).

By embracing a data‐driven, adaptive management framework, conservation programmes can better support these desert ungulates to not only persist but also thrive in the long run (Armstrong and Seddon 2008; IUCN/SSC 2013). The challenge ahead will be to expand these approaches to more species and sites. This will require addressing persistent data gaps and mitigating external threats, so that density‐dependent regulation can be used as a tool to inform population recovery. Improving our understanding of ungulate dynamics in arid environments will strengthen conservation planning and support more sustainable outcomes, ultimately helping to secure resilient and growing populations of Arabian oryx, sand gazelles, and other arid‐land ungulates for the future (Mace et al. 2018; Seddon et al. 2014).

## Author Contributions

**Ahmed S. Aljohani:** conceptualization (lead), data curation (lead), formal analysis (lead), funding acquisition (lead), investigation (lead), methodology (lead), writing – original draft (lead), writing – review and editing (equal). **Michael B. Bonsall:** conceptualization (equal), data curation (supporting), formal analysis (supporting), methodology (supporting), project administration (equal), resources (equal), supervision (equal), validation (equal), writing – review and editing (equal). **Robert A. Montgomery:** conceptualization (equal), formal analysis (supporting), methodology (supporting), project administration (equal), supervision (equal), validation (equal), writing – review and editing (equal).

## Funding

A.S.A. was supported by the Saudi Arabian Cultural Bureau and the Rhodes Trust.

## Disclosure

Statement of inclusion: Our study was designed and led by a Saudi PhD student with co‐authors based in the UK, combining regional knowledge with quantitative expertise. To enhance inclusivity, the team collaborated from the outset to ensure diverse perspectives and appropriate contextual interpretation. Regional literature and data sources were cited whenever possible, and guidance was sought from scientists based in the Middle East, including colleagues in Egypt and Iran.

## Conflicts of Interest

The authors declare no conflicts of interest.

## Data Availability

Data supporting this study are archived in a public repository following this link: https://osf.io/ydrzn/overview?view_only=a7f7d5bf22f04a50aee5a3f6e0e1bfb6.
